# Identifying overcontrol and undercontrol personality types among young people using the five factor model, and the relationship with disordered eating behaviour, anxiety and depression

**DOI:** 10.1186/s40337-024-00967-4

**Published:** 2024-01-24

**Authors:** Tanya Gilmartin, Joanna F. Dipnall, Caroline Gurvich, Gemma Sharp

**Affiliations:** 1 Department of Neuroscience, Monash University and the Alfred Hospital, Melbourne, Australia; 2 School of Public Health and Preventive Medicine, Monash University, Melbourne, 3004 Australia; 3 Institute for Mental and Physical Health and Clinical Translation, School of Medicine, Deakin University, Geelong, 3220 Australia; 4 Department of Psychiatry, HER CENTRE Australia, Central Clinical School, Monash University, Melbourne, Australia

**Keywords:** Eating disorders, Disordered eating, Personality types, Overcontrol, Undercontrol, Resilient

## Abstract

**Background:**

Overcontrol and undercontrol personality types have been associated with an increase in eating pathology, depression and anxiety. The aim of the research was to explore whether latent overcontrol and undercontrol personality types could be identified using cluster analysis of the facets of the five factor model (FFM). We further aimed to understand how these personality types were associated with eating pathology, depressed mood and anxiety.

**Methods:**

A total of 561 participants (394 women and 167 men), aged 16–30 years in Australia completed a survey designed to assess disordered eating, FFM personality traits, anxiety, depression and stress. A systematic four-step process using hierarchical, k-means, and random forest cluster analyses were used to identify a meaningful 3-cluster solution.

**Results:**

The results revealed a cluster solution that represented overcontrol, undercontrol and resilient personality types, and highlighted facets of the FFM that were associated with each type. Both overcontrol and undercontrol personality types were associated with increased clinical symptoms compared to the resilient types.

**Conclusions:**

It was concluded that FFM facets may potentially be more meaningful than broad domains in identifying personality types, and that both overcontrol and undercontrol personality types are likely associated with increased clinical symptoms.

**Supplementary Information:**

The online version contains supplementary material available at 10.1186/s40337-024-00967-4.

## Abstract (plain language summary)

Personality has previously been found to be strongly related to eating disorders and disordered eating. A person’s personality is made up by a series of personality traits. A series of traits grouped together is called a personality type. Three broad personality types have been identified consistently in previous research, being overcontrol (rigid, perfectionistic), undercontrol (impulsive, mood dependent) and resilient (flexible, low pathology). Understanding eating disorders in the context of overcontrol, undercontrol and resilient personality types has been found to be predict disordered eating behaviour and treatment course for people with eating disorders. This study aimed to identify personality types using the well known five factor model of personality among a group of Australian young people. We also aimed to understand the relationships between personality type and eating pathology. It was found that a wider range of personality traits may be more meaningful than broad domains in identifying personality types. It was further found that individuals who were overcontrol or undercontrol were more likely to experience eating pathology and disordered eating compared to resilient types. Overall, this means that that the five factor model of personality may be useful for identifying people at risk for developing an eating disorder and to consider treatment needs.

**Supplementary Information:**

The online version contains supplementary material available at 10.1186/s40337-024-00967-4.

## Background

Personality and personality pathology are domains that have attracted research attention in order to elucidate why some people may be more vulnerable to developing a clinical disorder [1–4]. Within this research it has been found that personality factors are associated with the development, course and maintenance of eating disorders (EDs), depression and anxiety disorders [4–8].

**Fig. 1:**
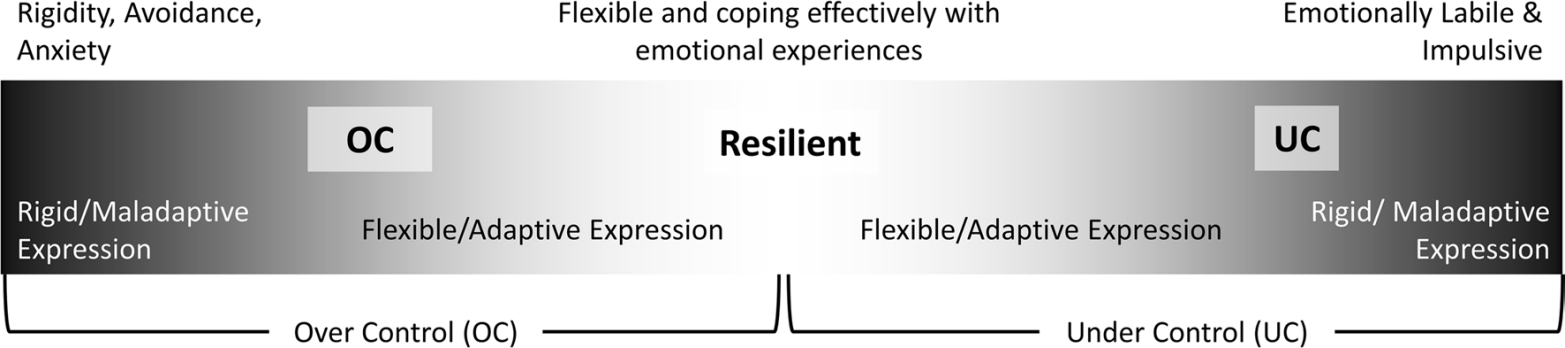
Overcontrol, undercontrol and resilient personality types

An area that has been extensively investigated is the relationship between personality types and psychopathology [1, 9, 10]. The developmental literature describes three personality types that form one bipolar dimension from overcontrol (OC) to undercontrol (UC) with a resilient type occupying the space in between (See Fig. 1 [11–14]). Both OC and UC personality types have been associated with a higher risk of developing an ED compared to resilient types [15] and comparable personality types are identified among patients presenting for treatment of depression [10]. Furthermore, classifying individuals who have EDs as OC, UC or resilient has been found to be more predictive of longitudinal outcomes in ED treatment (Described in more detail below; [16, 17]).

There has been increasing criticism of the categorical model of diagnostic classification employed by the diagnostic manuals such as the diagnostic and statistical manual of mental disorders (DSM-5; [18]) citing high co-occurrence of mental health concerns, diagnostic instability and a lack of specificity in treatment [19]. As a result, researchers have sought alternative frameworks to better understand psychopathology (e.g. [19]). One argument put forward is that that classifying clinical disorders based on personality types may offer an advantage over current categorical diagnoses as they are transdiagnostic and stable over time [1, 16, 19, 20]. For example, there is a high instance of co-occurrence between eating disorders, anxiety and depression [21–24], with evidence that anxiety will emerge before the development of an eating disorder [21]. Therefore conceptualising individual difficulties based on personality type may increase the understanding of the precipitating and maintaining factors for not just eating disorders in isolation, but also for other high prevalence concerns. In their systematic review, Bohane and team [9] noted that in spite of the promise offered by classifying EDs based on types, a considerable barrier is that there is no clear consensus of how to understand and assess personality types, which limits the translation of the current literature into clinical practice.

### Defining personality types from over to under control

In order to further understand the relationship between personality and disordered eating, we will review the current understanding of the traits that define each personality type. The OC group has been described as rigid, compulsive and as having difficulties with intimacy, as well as poor self-esteem, anxiousness, hypersensitivity and introversion [9, 16, 25–27]. Personality pathology associated with maladaptive OC presentations include obsessive–compulsive, paranoid, avoidant and schizoid personality disorders [9, 28]. The OC type has been identified in samples of individuals who have been diagnosed with an eating disorder [16, 25–27] and non-clinical samples [10, 29]. The OC type has been found to be more common among individuals with anorexia nervosa-restricting subtype and atypical anorexia nervosa [9, 30], and higher levels of depression have been identified among individuals with PTSD compared to the UC type [9, 31]. Research has indicated that OC patients with EDs respond better to intensive treatment interventions than UC patients (described below; [16, 32].

In contrast, the UC type has a tendency to express emotion inappropriately, engage in risk taking behaviours and are associated with externalising problems [13]. UC has been associated with features characteristic of borderline personality pathology specifically such as self-injurious behaviour, impulsivity and poor affect regulation [9, 16, 25, 27, 28, 33]. This group has been identified in groups of people with varied ED diagnoses [26, 27] in addition to samples consisting only of patients with anorexia nervosa, bulimia nervosa, and non-clinical samples [10, 16, 25, 26, 29]. The UC type has been found to be associated with higher incidence of bulimia nervosa [9, 30] compared to the OC type. Individuals presenting with anorexia nervosa within the UC type have been found to have a poorer initial response to intensive treatment and an elevated risk of readmission within 3 months of discharge [16].

The low-psychopathology or resilient type is considered to be well-adjusted [34], and cope effectively with difficulties that they may face. Further this type is characterised by resourceful adaptation to changing circumstances and flexible use of problem-solving strategies [35]. In samples of individuals with eating disorders, this group has been described as perfectionistic and high functioning [27], or as presenting without personality pathology [9, 16, 25, 26]. Lynch [28] defines the resilient group as adaptive variants of OC and UC that can flexibly respond to a variety of stimuli. Individuals within the resilient group are not immune to the development of mental health concerns, as a resilient or high functioning group has been identified in all studies examining personality types in eating disorders [16, 25, 26, 30, 32, 36]. However, research in non-clinical samples indicate lower incidence of EDs in the resilient group compared to the OC or UC groups [29].

### Five factor model

The five factor model (FFM) theory posits there are five broad domains of normative personality that succinctly describe an individual’s style of thinking, feeling and interacting; Neuroticism, Extraversion, Openness, Agreeableness and Conscientiousness. Within each domain their resides six facets (For further description, see: [37–40]). Previous studies have used the FFM to explore and understand personality types. Within this research, both OC and UC types have been associated with higher Neuroticism [15, 41, 42], but the UC type has also been associated with low Agreeableness and Conscientiousness, and the OC type with low Extraversion, Openness [15, 41, 42] and both high [41, 42] and low Conscientiousness [15]. The resilient or high-functioning group has been associated with low Neuroticism and higher than average scores on the other domains [15, 41, 42].

Although the FFM has been frequently used to understand personality types in previous research, there continue to be barriers to translating such research into clinical practice. Previous studies have relied on broad FFM domains as the measure on which to base personality types; however, examining personality facets within the five broad domains can provide more information [37, 38, 40, 43]. Secondly, most previous studies have clustered participant data within clinical samples. A concern may be that this strategy might result in skewed data. For example, traits that exemplify OC or UC when comparing groups from a solely eating disorders sample who as an overall group score higher than the general population on measures of anxiety and perfectionism [21, 44–46], may differ from traits that exemplify personality types when comparing non-clinical groups. Drawing data from a non-clinical sample might present a less biased representation of the OC/UC spectrum. Thirdly, although most clinical samples only consist of girls or women participants [16, 25–27], and girls/women have been found to be more likely to develop an ED compared to boys/men [18, 47], disordered eating has been increasing at faster rate among men compared to women [48, 49]. In the limited research on EDs or disordered eating among men, gender differences have been found between personality and disordered eating [43, 50]. Therefore, it was considered important within the design of our study to include men, and to control for gender in our analyses.

### The present study

Our study had two aims. The first was to explore whether clusters exist in a mixed gender, community sample that represent OC, UC and resilient personality types. It was expected that distinct latent clusters of participants who were characterised by traits that can be compared with previously identified OC, UC and resilient types would be distinguishable in the data.

Secondly, we aimed to explore how cluster membership predicted eating pathology, depression, anxiety and stress. We expected that the OC and UC types would score higher on measures of disordered eating, depression, anxiety and stress compared to resilient types. The broader aim of this research was to improve the understanding of conceptualising eating and clinical pathology in the context of personality types.

## Methods

### Participants

All procedures were approved by Monash University Human Research Ethics Committee prior to study commencement. Out of a prospective 1,492 individuals, 572 individuals completed all measures of the study. A flowchart of participation and study drop-out has previously been published [50]. Prospective participants accessed information about the study from an online advertisement. The ages of participants ranged from 16 to 30 (M = 22.15, SD = 3.84), including 167 men (29.2%, M = 21.76, SD = 3.62), 395 women (69.1%, M = 22.32, SD = 3.95) and 10 individuals identified as gender diverse (1.8%, M = 21.8, SD = 3.05). The sample was mainly Caucasian/White (70%) and/or had a Year 12 (final year of high school) or equivalent education (45%). Our sample has been described in more detail in our previous work [50] and sufficient statistical power has been found to be adequate in relatively small samples for cluster analysis [51].

### Measures

Participants completed a survey designed to measure FFM personality facets and domains, and eating behaviour. All measures used in the current study have been validated in comparable samples [52–56].

#### Personality

The FFM International Personality Item Pool-Neuroticism Extraversion Openness scale-120 item version (IPIP-NEO-120; [52]) was used as a measure of the five domains (Neuroticism, Extraversion, Openness, Agreeableness and Conscientiousness) and 30 facets of the FFM. Participants were asked to rate how each item described them (e.g., “Love excitement,” “Avoid philosophical discussions”) on a five-point Likert scale ranging from 1 (Very Inaccurate) to 5 (Very Accurate). To obtain facet scores, the four items associated with each facet are summed. Internal consistency was found to be inadequate for the E6 (Cheerfulness; α = 0.56) and O4 (Adventurousness; α = 0.58) subscales, so these scales were removed from subsequent analyses. The E5 (Excitement Seeking; α = 0.68) and O3 (Emotionality; α = 0.66) subscales were included in the study based on contemporary references that indicate that internal consistency may be acceptable [57]. The remainder of the subscales demonstrated adequate to strong internal consistency (α = 0.70–0.90). The IPIP-NEO-120 has been found to provide consistent results from measurement of FFM domains and facets with the original IPIP-NEO [58] and the NEO-PI-R, the measure on which it was based [52]. The five-factor structure of the IPIP-NEO has been replicated across samples [59], and the NEO, which the IPIP-NEO-120 was based on, has been found to be invariant across genders [60].

#### Eating behaviour

We assessed disordered eating behaviour using the eating pathology symptoms inventory (EPSI; [61]). This is a 45-item scale designed to assess disordered eating behaviours. The scale consists of eight subscales, with body dissatisfaction providing a measure of core eating and weight concerns, and seven subscales designed to measure specific disordered eating behaviours. The Restriction scale measures a tendency to restrict dietary intake, the Binge eating scale assesses the tendency to ingest large amounts of food, the Purging subscale measures self-induced vomiting, laxative use, diuretic use, and diet pill use. The Cognitive Restraint scale measures calorie counting and a focus on “healthy” foods and Negative Attitudes Towards Obesity measures judgements about individuals who were perceived as overweight. The Excessive Exercise subscale provides an assessment of compulsive or intense exercise and the Muscle Building subscale assesses efforts to build muscle and supplement use. An additional item was included as part of the scale to measure chewing and spitting behaviour (“I spat out food after chewing to avoid putting on weight”) based on surveys administered by Aouad and his team [62] and worded to remain consistent with the other items in the EPSI. The EPSI is scored on a five-point Likert scale ranging from 0 (Never) to 4 (Very often), and the items for each subscale are summed together to obtain a total score. The internal consistency in the current study was found to range from good to strong (α = 0.79–0.90). The EPSI has previously been found to have good test–retest reliability for all scales for men and women together, in addition to being invariant across gender [61], and for most scales when genders were considered separately [63].

The eating disorder examination questionnaire-short (EDE-QS) has been developed as a 12-item version of the original eating disorder examination questionnaire (EDE-Q; [64]), and the scale asks participants to select on how many days they engaged in particular behaviours (e.g., “Have you had a strong desire to lose weight?” “Have you had a sense of having lost control over your eating”) with items are scored on a 4-point Likert scale ranging from 0 (0 days) to 3 (6–7 days). The scores obtained on each item are added together to achieve a total score [64]. Research has indicated that scores of 15 and above are indicative of the presence of an eating disorder [65]. The scale has been found to have strong internal consistency, with a Cronbach’s alpha coefficient of α = 0.91 in the current sample. In addition, the EDE-QS has been found to have high test–retest reliability over 2–14 days, and strongly correlated with the EDE-Q and other measures of eating pathology indicating strong convergent validity, and the scale has been found to effectively discriminate between individuals with eating disorders and those without [64]. The EDE-Q has been found to be appropriate for use with males and females [66].

#### Depression, anxiety and stress

The depression anxiety stress scale-21 item version (DASS-21) is a 21-item scale that is used to measure negative mood states. The scale was derived from the original 42-item DASS, and consists of three subscales [67]. The first, Depression, measures low positive affect (e.g., “I couldn't seem to experience any positive feeling at all”), Anxiety measures physical hyperarousal (e.g., “I felt I was close to panic”) and Stress measures tension or irritability (e.g. “I found it difficult to relax”; [68]). The DASS-21 is scored on a 4-point Likert scale ranging from 0 (Did not apply to me at all/Never) to 4 (Applied to me very much or most of the time/Almost Always), with the item scores for each subscale summed to obtain total scores, and then multiplied by two to obtain scores consistent with the original 42-item version [67]. Research has replicated the three-factor structure of the DASS and DASS-21 [67, 68] and the DASS has demonstrated good internal consistency in our sample (α = 0.84–0.91).

### Procedures

Data from this study have previously been described elsewhere [50, 69]. In order to attract prospective participants, advertisements providing a brief overview of the goals of the study and anticipated time commitment were posted on social media. The posts were targeted towards young adults and were posted on social media pages and electronic newsletters associated with eating disorder and personality disorder organisations, university pages, community and sport notice boards, and pages associated with interest groups such as fitness, trades and food. The survey consisted of questions to collect demographic information and psychometric measures. At the completion of the survey, participants were provided with the opportunity to enter a draw to win a voucher worth the equivalent of $35 USD.

### Statistical analysis

**Fig. 2:**
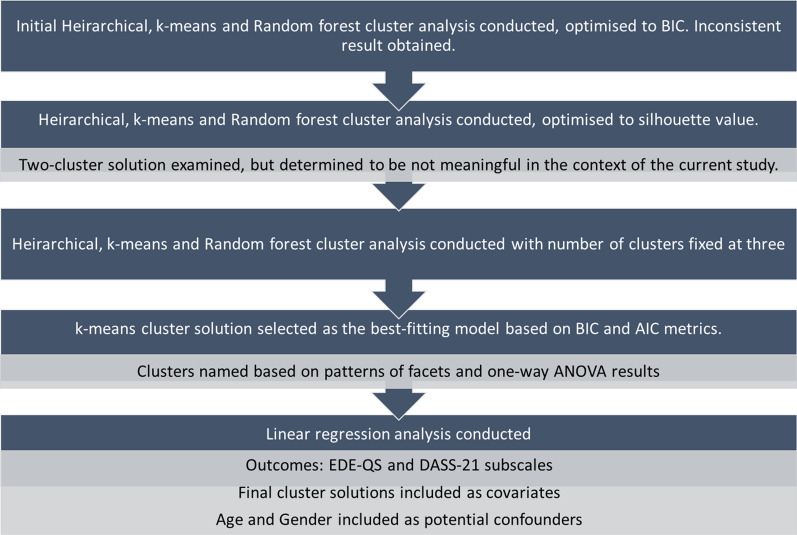
Data analysis flowchart

Cluster analyses were conducted using JASP statistical package version 0.14.1 (Jasp Team, [70]), and all other statistical calculations were implemented using SPSS version 27 [71]. The JASP user manual is freely available online [72], and the machine learning parameters used in our study have been included in the Additional file 1. The statistical analysis process has been outlined as Fig. 2. After E6 (Cheerfulness) and O4 (Adventurousness) were removed due to poor internal consistency, all other IPIP-NEO-120 personality facets were entered into Hierarchical, k-means, and random forest cluster analyses. The silhouette value [73] in addition to the Akaike Information Criterion (AIC; [74]) and Bayesian Information Criterion (BIC; [75]) were used to determine the most suitable number of clusters.

The naming of the final cluster solution from the models was informed from the pattern of means and standard deviations of the input variables. In particular, the differences between clusters on IPIP-NEO personality facets, EPSI and DASS-21 subscale scores and EDE-QS were examined using one-way Analysis of Variance (ANOVAs), the frequency of clinical significance of EDE-QS scores were compared between clusters using a chi-squared analysis, and linear regression analyses were used to explore how cluster membership would predict EDE-QS and DASS-21 scores. The gender and age of the respondent were included as potential confounders in the regression. Age was included in the analysis because eating disorders are more likely to emerge in adolescence than adulthood, and individuals younger in age have been found to be more likely to restrict eating [18, 50].

## Results

Preliminary analyses including the means and standard deviation for each of the variables have been included in our Additional file 1. A correlation matrix of all variables has previously been published within Additional file 1: Data for our previous publication [50].

For the initial cluster analysis, we optimised the solution to the silhouette value for the hierarchical, k-means, and random forest methods. For each method, a 2-cluster solution appeared to be the best fitting solution: Cluster 1 demonstrated high scores on facets within Neuroticism, and low scores on facets within Extraversion and Conscientiousness; and Cluster 2 demonstrated low scores on facets within Neuroticism, and high scores on facets within Extraversion and Conscientiousness (see Additional file 1). Differences between EPSI and DASS-21 scores from the two-cluster solutions reflected high compared to low psychopathology clusters. However, since the aim of the current study was to explore potential facet-level differences, and to understand the nuances of maladaptive personality presentations, a 3-cluster solution was used for further analysis. The revised number of clusters was based on the examination of the dendrogram (see Additional file 1) in addition to the existing research literature which has demonstrated at least two different forms of high psychopathology personality presentations [9, 16, 25–27].

Hierarchical, k-means, and random forest cluster analyses were then conducted with the number of cluster solutions fixed at three. The Akaike Information Criterion (AIC; [74]) and Bayesian Information Criterion (BIC; [75]) were used to select the k-means 3-cluster solution to use for further statistical analysis. The AIC and BIC are commonly used as model selection criterions, where the lowest value represents the better model [76, 77].

Table 1 summarises the number of participants allocated to each cluster for the Hierarchical, k-means, and random forest cluster analyses three cluster solutions in addition to the BIC and AIC values.

**Table 1:** Summary of Cluster solutions, and number of males, females and gender diverse participants in each cluster for each cluster solution in addition to BIC and AIC values

	Method		
	Hierarchical	k-means	Random forest
*Overcontrol (OC)*			
Men	76 (21.8%)	37 (19.3%)	72 (21.7%)
Women	266 (76.4%)	151 (78.6%)	253 (76.2%)
Gender diverse	6 (1.7%)	4 (2.1%)	7 (2.1%)
Total	348	192	332
*Undercontrol (UC)*			
Men	49 (50.0%)	59 (35.1%)	52 (34.9%)
Women	47 (48.0%)	105 (62.5%)	95 (63.8%)
Gender diverse	2 (2.0%)	4 (2.4%)	2 (1.3%)
Total	98	168	149
*Resilient*			
Male	42 (33.3%)	71 (33.5%)	43 (47.3%)
Female	82 (65.1%)	139 (65.6%)	47 (51.6%)
Gender diverse	2 (1.6%)	2 (0.9%)	1 (1.1%)
Total	126	212	91
BIC	13,410.56	**13,111.33**	13,757.06
AIC	13,058.28	**12,746.00**	14,122.38

E6 (Cheerfulness) and O4 (Adventurousness) were removed due to questionable internal consistency, **Bold** denotes lower BIC and AIC values

### Final cluster solutions

The k-means cluster analysis had the lowest BIC and AIC values, and was therefore selected as the final cluster solution for the following analysis. The means and standard deviations for the Hierarchical and Random forest cluster analyses have been included as Additional file 1. A series of one-way Analysis of Variances (ANOVAs) and Bonferroni adjusted Post Hoc analyses were conducted to identify the IPIP-NEO-120 facets that distinguished each cluster. Table 2 displays the means and standard deviations, for the IPIP-NEO-120, EPSI, EDE-QS and DASS-21 and ANOVA results for between cluster comparisons for the final 3-cluster solution.

**Table 2:** Means, standard deviations and one-way ANOVA results for the IPIP-NEO-120 facets, EPSI, EDE-QS and DASS-21 by final 3-cluster solution

Subscale	Overcontrol (OC) N = 192		Undercontrol (UC) N = 168		Resilient N = 212		F(2,569)	Partial eta^2^
	M	SD	M	SD	M	SD		
*IPIP-NEO-120 Neuroticism*								
N1: Anxiety	**17.32**^**cd**^	3.61	14.75^ed^	2.52	12.35^ec^	4.10	102.31**	0.26
N2: Anger	11.73^cd^	4.10	**13.63**^**ed**^	4.38	9.42^ec^	3.75	50.68**	0.15
N3: Depression	**15.09**^**cd**^	3.67	13.39^ed^	3.68	9.28^4ec^	3.98	126.07**	0.31
N4: Self-consciousness	**16.71**^**cd**^	2.96	13.23^ed^	2.15	12.17^ec^	3.11	144.02**	0.34
N5: Immoderation	12.46^cd^	3.55	**13.73**^**ed**^	3.81	10.24^ec^	3.34	47.11**	0.14
N6: Vulnerability	**15.73**^**cd**^	3.36	13.62^ed^	2.67	10.41^ec^	3.38	146.31**	0.34
*IPIP-NEO-120 extraversion*								
E1: Friendliness	9.65^cd^	3.07	12.02^ed^	3.12	**14.92**^**ec**^	2.81	157.34**	0.36
E2: Gregariousness	7.59^ec^	3.87	**11.65**^**c**^	2.83	**12.11**^**e**^	3.69	98.71**	0.26
E3: Assertiveness	10.22^cd^	3.37	12.46^ed^	3.38	**14.29**^**ec**^	2.98	79.84 **	0.22
E4: Activity	11.07^c^	3.00	11.23^e^	3.24	**13.62**^**ec**^	3.13	41.76**	0.13
E5: Excitement seeking	9.73^cd^	2.89	**13.43**^**ec**^	2.84	11.33^ec^	2.72	77.61**	0.21
*IPIP-NEO-120 openness*								
O1: Imagination	13.80^c^	3.46	**15.09**^**ce**^	3.91	13.25^e^	3.76	11.79**	0.04
O2: Artistic interests	13.64^c^	3.41	13.42^e^	3.59	**14.67**^**ec**^	3.63	7.00*	0.02
O3: Emotionality	**15.13**^**c**^	3.42	13.67^ec^	3.43	**14.97**^**e**^	3.18	10.16**	0.03
O5: Intellect	13.98^c^	3.37	13.88^e^	3.54	**16.10**^**ec**^	2.83	30.00**	0.10
O6: Liberalism	**13.85**^**e**^	2.98	13.00^e^	3.07	13.65	3.28	3.58*	0.01
*IPIP-NEO-120 agreeableness*								
A1: Trust	11.90^c^	3.44	11.91^e^	3.77	**15.14**^e**c**^	3.06	59.78**	0.17
A2: Morality	**18.35**^**c**^	2.76	14.57^ec^	1.92	**18.21**^**e**^	2.04	162.73**	0.36
A3: Altruism	16.68^cd^	2.85	14.45^ec^	2.54	**17.56**^**ec**^	2.20	73.95**	0.21
A4: Cooperation	**16.95**^**c**^	3.05	13.10^ec^	2.70	**17.58**^**e**^	2.38	145.14**	0.34
A5: Modesty	**17.34**^**ec**^	3.89	13.34^c^	2.81	14.01^e^	3.54	73.14**	0.21
A6: Sympathy	**16.24**^**c**^	2.94	13.98^ec^	2.76	**16.38**^**e**^	2.94	39.18**	0.12
*IPIP-NEO-120 conscientiousness*								
C1: Self-efficacy	13.22^c^	2.76	13.46^e^	2.79	**16.81**^**ec**^	1.93	129.81**	0.31
C2: Orderliness	12.88^cd^	4.06	11.53^ec^	4.32	**14.73**^**ec**^	3.76	30.16**	0.10
C3: Dutifulness	16.82^cd^	2.75	13.63^ec^	2.10	**17.55**^**ec**^	1.79	160.54**	0.36
C4: Achievement striving	13.90^cd^	3.04	12.71^ec^	3.26	**16.97**^**ec**^	2.52	108.70**	0.28
C5: Self-discipline	10.91^c^	2.69	10.61^e^	3.10	**14.53**^**ec**^	2.69	118.27**	0.29
C6: Cautiousness	15.07^cd^	3.40	10.68^ec^	3.78	**15.90**^**ec**^	2.97	123.71**	0.30
*EPSI*								
Body dissatisfaction	**17.08**^**c**^	6.97	**16.36**^**e**^	7.26	11.27^ec^	7.00	40.35**	0.12
Binge eating	13.14^cd^	7.47	**15.44**^**ec**^	7.93	9.84^ec^	6.76	27.76**	0.09
Cognitive restraint	5.62	3.46	5.60	3.44	5.05	3.40	1.79	0.01
purging	2.99^cd^	5.76	**4.28**^**ec**^	5.07	1.28^ec^	3.12	19.60**	0.06
Restriction^a^	**8.95**^**c**^	6.02	**9.95**^**e**^	6.21	7.07^ec^	6.08	11.00**	0.04
Excessive exercise^a^	6.59^e^	5.34	**8.23**^**e**^	5.35	7.26	5.45	4.20*	0.02
Negative attitudes towards obesity	5.83^c^	5.36	**8.40**^**ec**^	5.36	5.55^e^	5.42	15.34**	0.05
Muscle building	3.23^c^	4.20	**4.51**^**ec**^	4.09	3.35^e^	3.82	5.40*	0.02
Chewing and spitting	0.25^c^	1.03	**0.55**^**ec**^	0.72	0.19^e^	0.70	10.00**	0.03
*EDE-QS and DASS-21*								
EDE-QS^b^	**13.20**^**c**^	9.03	**14.82**^**e**^	9.00	8.81^ec^	7.78	25.46**	0.08
DASS-21 depression	**22.72**^**c**^	11.82	**21.35**^**e**^	12.23	9.71^ec^	9.96	80.12**	0.22
DASS-21 anxiety	**18.83**^**c**^	10.87	**18.39**^**e**^	11.12	9.57^ec^	10.11	48.10**	0.15
DASS-21 stress	**22.07**^**c**^	10.21	**21.63**^**e**^	10.00	13.55^ec^	10.00	45.69**	0.14

Matching subscripts within the same row (c, d, e) denote significant differences as identified in the Bonferroni adjusted Post Hoc analyses. Bold denotes highest value (*p* < 0.05)

*p < 0.05, **p < 0.001

^a^ANOVA is F(2,568)

^b^ANOVA is F(2,566)

As demonstrated in Table 2, three clusters emerged from the data that resembled previously identified theoretical constructs. An OC-like cluster was identified that was characterised by high anxiety, depression, self-conscientiousness, vulnerability, emotionality, liberalism, morality, cooperation, modesty and sympathy. The OC cluster was further characterised by low scores on facets within the Extraversion domain and scores on facets within the Conscientiousness domain that were significantly higher than the UC group, but significantly lower than the Resilient group. The UC-like group were found to have high anger, immoderation, gregariousness, excitement seeking, imagination and low scores on facets within the Agreeableness and Conscientiousness domains. Significantly higher scores on EPSI Body dissatisfaction, restriction DASS subscales and EDE-QS were found in both the OC and UC groups compared to the resilient groups, while the UC group were uniquely characterised by high scores on EPSI Binge eating, purging, negative attitudes towards obesity and chewing and spitting. A resilient cluster was clearly identifiable that was characterised by low scores on facets within the Neuroticism domain and high scores on facets within the Extraversion, Agreeableness and Conscientiousness domains.

### Predicting eating pathology

Scores of 15 or above on the EDE-QS have been found to be indicative of the potential presence of an eating disorder [65]. The frequencies of participants who achieved a clinical EDE-QS score by cluster have been displayed in Table 3. Three participants had completed that IPIP-NEO-120 but not the EDE-QS and were therefore included in the cluster analyses but excluded from the chi-squared analysis. A chi-square test of independence was performed to examine the relationship between cluster solution and clinical EDE-QS score. The relationship was found to be significant (Χ^2^(N = 569) = 43.57, *p* < 0.001). Individuals in the OC and UC clusters were found to be more likely to achieve clinical scores on the EDE-QS than those in the resilient cluster.

**Table 3:** Frequency and percentage of clinical EDE-QS score by personality type

Cluster	N	EDE-QS score < 15 N (%)	EDE-QS score ≥ 15 N (%)
Overcontrol (OC)	191	107 (56%)	84 (44%)
Undercontrol (UC)	168	80 (47.6%)	88 (52.4%)
Resilient	210	166 (79%)	44 (21%)

N = 569

Results of the linear regression analyses to explore the cluster IPIP-NEO cluster solutions being a predictor of eating pathology are presented in Table 4

**Table 4:** Results of linear regression analysis using 3-cluster membership to predict eating pathology, depression, anxiety and stress

	Predictor	b	SE	beta	t	*p* value	Part r	Partial r
*EDE-QS*								
Block 1	(Constant)	8.81	0.59		14.88	< 0.001		
	OC^a^	4.40	0.86	0.23	5.12	< 0.001	0.21	0.21
	UC^a^	6.01	0.89	0.31	6.77	< 0.001	0.27	0.27
Fit	R^2^ = 0.08, F(2,566) = 25.46, *p* < 0.001							
Block 2	(Constant)	7.94	2.18		3.64	< 0.001		
	OC^a^	3.86	0.85	0.20	4.56	< 0.001	0.19	0.18
	UC^a^	6.08	0.87	0.31	6.97	< 0.001	0.28	0.28
	Age	− 0.08	0.09	− 0.03	− 0.83	0.408	− 0.04	− 0.03
	Female^b^	3.98	0.79	0.21	5.06	< 0.001	0.21	0.20
	Gender diverse^b^	− 1.25	2.74	− 0.02	− 0.46	0.648	− 0.02	− 0.02
Fit	R^2^ = 0.12, F(5,563) = 16.26, *p* < 0.001							
Difference	ΔR^2^ = 0.04							
*DASS-21 Depression*								
Block 1	(Constant)	9.71	0.78		12.46	< 0.001		
	OC^a^	13.02	1.13	0.48	11.52	< 0.001	0.43	0.44
	UC^a^	11.65	1.17	0.42	9.94	< 0.001	0.37	0.39
Fit	R^2^ = 0.22, F(2,566) = 80.12, *p* < 0.001							
Block 2	(Constant)	9.62	2.94		3.27	0.001		
	OC^a^	13.07	1.14	0.48	11.43	< 0.001	0.43	0.43
	UC^a^	11.69	1.18	0.42	9.92	< 0.001	0.37	0.39
	Age	0.01	0.12	0.00	0.07	0.944	0.00	0.00
	Female^b^	− 0.13	1.06	− 0.01	− 0.13	0.900	− 0.01	− 0.01
	Gender diverse^b^	− 2.51	3.70	− 0.03	− 0.68	0.497	− 0.03	− 0.03
Fit	R^2^ = 0.21, F(5,563) = 32.00, *p* < 0.001							
Difference	ΔR^2^ = 0.00							
*Dass-21 anxiety*								
Block 1	(Constant)	9.57	0.73		13.04	< 0.001		
	OC^a^	9.27	1.06	0.38	8.71	< 0.001	0.34	0.34
	UC^a^	8.83	1.10	0.35	8.00	< 0.001	0.31	0.32
Fit	R^2^ = 0.14, F(2,569) = 48.10, *p* < 0.001							
Block 2	(Constant)	13.75	2.76		4.99	< 0.001		
	OC^a^	8.67	1.11	0.34	7.85	< 0.001	0.30	0.31
	UC^a^	9.04	1.07	0.37	8.43	< 0.001	0.33	0.33
	Age	− 0.22	0.12	− 0.08	− 1.92	0.056	− 0.07	− 0.08
	Female^b^	1.23	1.00	0.05	1.23	0.218	0.05	0.05
	Gender diverse^b^	2.24	3.48	0.03	0.64	0.520	0.03	0.03
Fit	R^2^ = 0.15, F(5,566) = 20.33, *p* < 0.001							
Difference	ΔR^2^ = 0.01							
*DASS-21 stress*								
Block 1	(Constant)	12.33	0.90		13.77	< 0.001		
	OC^a^	3.71	1.35	0.13	2.74	0.006	0.11	0.11
	UC^a^	9.56	1.05	0.43	9.14	< 0.001	0.36	0.36
Fit	R^2^ = 0.14, F(2,569) = 46.19, *p* < 0.001							
Block 2	(Constant)	6.83	2.67		2.55	0.011		
	OC^a^	4.24	1.36	0.15	3.12	0.002	0.12	0.13
	UC^a^	9.41	1.05	0.43	8.97	< 0.001	0.35	0.35
	Age	0.18	0.11	0.06	1.60	0.110	0.06	0.07
	Female^b^	2.29	0.95	0.10	2.40	0.017	0.09	0.10
	Gender diverse^b^	2.46	3.26	0.03	0.75	0.451	0.03	0.03
Fit	R^2^ = 0.15, F(5,566) = 20.46, *p* < 0.001							
Difference	ΔR^2^ = 0.01							

*b* unstandardized regression estimate, *beta* standardized regression estimate, *SE* standard error, *r* correlation coefficient

^a^Reference category = Resilient group

^b^Reference category = being male

As displayed in Table 4, being in the OC or UC cluster was found to significantly predict a higher score on the EDE-QS and all three DASS-21 subscales compared to membership in the resilient cluster. All of these relationships remained significant when the influence of age and gender was considered. Being a woman was found to add significant value to the predictive model compared to being a man for the EDE-QS and the Stress DASS-21 subscale.

## Discussion

The purpose of our study was to explore the relationship between personality types and eating pathology in a young adult non-clinical sample. As the objective of this study was to understand facet-level differences between high psychopathology groups, we selected a three-cluster solution as the most meaningful in the context of past research and goals of the study. As expected, OC, UC and resilient personality clusters were distinguishable in our study data. Here, we explore in detail the facets that were related to each personality type to strengthen the understanding of the factorise that characterise OC, UC and resilience. We then discuss how disordered eating, depression, anxiety and stress differed between personality type.

### Personality types

Supporting the hypotheses of the current study, the OC type was associated with high scores on facets within the Neuroticism, Agreeableness and Conscientiousness domains and low scores on facets within the Extraversion domain. The UC type was associated with high scores on facets in the Neuroticism domain, and low scores on Agreeableness and Conscientiousness, and the resilient type was associated with low Neuroticism, and high Extraversion and Agreeableness [15, 41, 42]. The current study further aimed to extend on previous research studies by exploring the facet-level differences between personality types. Within the Neuroticism domain, the OC type was associated with high depression, anxiety, self-consciousness and vulnerability, while the UC type scored higher on anger and impulsivity. The results of the present study were also able to provide further insight into the relationship between personality types and the Conscientiousness domain. Our results indicated that the resilient type scored highest on all facets within the Conscientiousness domain. However, the OC type scored higher than the UC group on all facets except self-efficacy and self-discipline.

While our study is the first to our knowledge to understand facet-level differences of personality types, the results reflect theoretical constructs of the OC type being anxious, sensitive and with low self-esteem [16, 27], in addition to placing a high importance on meeting commitments and high risk sensitivity [28]. On the other hand, impulsivity, aggression and externalisation have been previously associated with UC [9, 13, 16, 33]. What might be implied is that facet-level differences may explain the inconsistencies in past research on the FFM and personality types that also supports other research into personality types.

Other facet-level relationships of note were that the OC type was characterised by being humble and direct. This finding may be indicative of clinical observations that the OC type have a tendency towards maladaptive variants of these traits such as being self-denigrating or blunt [28]. An interesting finding was that the OC type scored high on the Liberalism (otherwise known as Values) FFM facet. Although not indicated in previous research, our findings may reflect a tendency of OC individuals to highly value rightness or fairness [28]. The UC type was associated with being imaginative, outgoing and adventurous, which may represent maladaptive tendencies to be unrealistic, attention seeking and reckless, which have previously been associated with the UC type [28]. Being resilient was found to be characterised by being trusting of others and strong with their own values. To consider the reverse of this finding, our research may reflect difficulties in relationships as experienced by both OC and UC, with limited trust in others and a tendency to be agreeable to the expense of their own values [28]. The important contribution of our research is that we can highlight the personality facets that may be indicative of personality type, which is over and above the current domain-level understanding.

### Personality types with disordered eating

The second aim of our study was to understand how personality type related to eating pathology. Although a clinical level of eating pathology was present among the resilient type, this was at a lower rate than their OC or UC counterparts, and both the OC and UC personality types were found to score equally high on measures of body dissatisfaction and dietary restriction compared to the resilient group. Membership in either the OC or UC cluster was found to predict the presence of eating pathology compared to the resilient type, as expected, supporting existing research [9]. The significance of the relationships remained when the influence of age and gender was controlled. Being a woman was found to also be a significant predictor of eating pathology and stress in predictive models, supporting past research that has highlighted identified gender differences in eating disorders [18, 47] and stress [78, 79].

There were some differences between the OC and UC types when considering disordered eating behaviours. The UC type scored higher than the OC type, and the OC type higher than the resilient type on measures of binge eating, purging, excessive exercise, muscle building and chewing and spitting. Although UC personality type has previously been linked to increased binge eating and purging behaviour [9, 30], the relationship between personality type and other eating behaviours has not been explored previously. For example, past research has identified a relationship between compulsive exercise and elevated Neuroticism [80, 81] and Extraversion [81], and chewing and spitting behaviour has previously been related to impulsivity [50, 69]. What can be implied is that elevated extraversion and impulsivity might put UC individuals at higher risk of compulsive exercise and chewing and spitting, respectively. There was little previous research exploring muscle building behaviour and FFM traits, so further research on this area is needed.

An interesting finding of our research was that UC participants scored higher on negative attitudes towards obesity compared to other personality types. While groups of “fat phobic” individuals with eating disorders have been identified in past research [82, 83], this has referred to a fear of weight gain in self rather than a judgement of the weight of others. Theoretically, OC individuals are more likely to internalise their concerns, while UC Individuals are more at risk of externalising behaviour [28]. Therefore, a speculative explanation may be that OC individuals may be more likely to internalise a fear of being in a larger body, whilst UC individuals are more likely to place expectation of body shape onto others, thus contributing to the perpetuation of weight stigma.

### Personality types with depression, anxiety and stress

Conceptualising clinical difficulties based on personality type theorised to be advantageous. As a transdiagnostic construct, there is capacity to understand the co-occurrence of clinical disorders as behavioural expressions of underlying personality [9, 28]. Therefore, we included measures of depression, anxiety and stress as a measure of clinical pathology, but also because of the high prevalence of these concerns among individuals who engage in disordered eating [21, 23]. Both the OC and UC types were found to score higher than the resilient types on measures of depression, anxiety and stress. There is previous research that has explored the relationship between personality and depression or anxiety [3, 4, 8, 84], and longitudinal research that has suggested that OC and UC are associated with higher rates of anxiety and depression [9]. However, to our knowledge, this is the first study that has explored the relationship between personality types and depression, anxiety and stress in a group of Australian young people. What can be implied is that OC and UC personality types are at higher risk of developing a mental health condition. An additional finding in the present study was that the UC type scored higher on measures of stress compared to the OC and resilient types. Our findings therefore provide a unique insight into the differences between OC and UC types is responding to their environment, which warrants further investigation.

To summarise the current findings, despite the differences in personality traits associated with each cluster, both were associated with similar clinical presentations. Theoretical frameworks outlined by Lynch [28] and Westen, Gabbard [85] suggest that the maladaptive interaction between an individual’s unique personality and the environment forms the building blocks to that individual’s psychopathology. Within these theoretical frameworks, our results suggest that although OC and UC personality types differ significantly, the psychopathology may appear outwardly comparable. Thus, our research provides further evidence that there may be some clinical utility in shifting the focus from the current diagnostic system that is focused on patterns of behaviour, to one where individual personality is the focus [20].

### Limitations

In interpreting the results of the current study, there are some limitations that should be considered. It is notable that the majority of participants who completed the survey for the current study identified as White. There is variability in eating disorder presentations between ethnic backgrounds [86] and between White and Indigenous Australians [87]. We also relied on self-report measures in this study, and had to omit some scales due to unacceptable internal consistency. Although using a large non-clinical sample is a strength in our research, it is also a limitation. This, alongside the use of a measure of normative personality are likely to create a barrier to the clinical translation of our research. It may be that a clinical version of the FFM [88] or a measure designed to assess maladaptive variants of FFM traits, such as the Personality Inventory for DSM-5 [89–92] may be more appropriate for clinical use.

The current data may be limited due to collection in Australia during the COVID-19 pandemic. The pandemic has been associated with increased psychological distress and eating disordered behaviours [93] and it is unclear if and when these factors will return to pre-pandemic levels. Finally, the current study’s cross-sectional design does not allow us to hypothesise how personality and eating behaviours may predict one another over time, but this study may provide a strong basis for a future longitudinal study for this purpose.

### Implications

The results of our study indicated that a facet-level understanding of personality types is likely to assist in understanding how to distinguish resilient types from OC or UC types using normative measures of personality. Secondly, our results extend on past research that has indicated that individuals with OC and UC personality types have increased eating pathology, depression, anxiety and stress compared to the resilient type, and that each personality type was associated with a range of disordered eating behaviour. Thirdly, comparable clinical behaviours may be overt representations of different underlying personality structure. For example, although an OC and a UC individual may both engage in restrictive eating behaviour or meet criteria for major depressive disorder, it may be implied that the factors that underlie the clinical presentation differ. Therefore, formulating an individual’s presenting pathology based on personality type may assist in understanding and addressing that individual’s unique concerns.

Our research has highlighted some important areas for future research. Firstly, the FFM is designed as a normative model of personality, where there are both adaptive and maladaptive representations of extreme scores and are also expected to remain stable over time [94]. In our study, it was evident that higher levels of psychopathology were associated with OC and UC types, and further research would benefit from understanding how to identify differences between adaptive and maladaptive representations of FFM traits. Although the current data was obtained using a non-clinical sample, it is possible to make speculative suggestions about future research in clinical samples. For example, further research is required to examine the FFM traits that are associated with each personality type, and how each of these traits predict treatment, and changes in presentation throughout treatment.

Another avenue of future research would be to explore how the FFM dimensions associated with OC and UC personality types relate to clinical conceptualisations of OC, UC and resilience. For example, Lynch has outlined that their treatment, Radically Open Dialectical Behaviour Therapy (RO-DBT) has some evidence as a treatment for maladaptive OC presentations [28], whereas Dialectical Behaviour therapy (DBT) has been designed for maladaptive UC personality styles [28, 95–98]. It would therefore be necessary to explore if the results of the current study are consistent with the outcomes of clinical interviews assessing for suitability for RO-DBT or DBT, and how this might compare with engagement with eating disorder specific treatment, such as Enhanced Cognitive Behaviour Therapy (CBT-E; [99]).

## Conclusions

The aim of the our study was to explore OC, UC and Resilient personality types using the FFM. The results revealed cluster solutions within the data that appeared consistent with the three personality types as theoretical constructs, and based on past research. Our results contributed to the research literature by creating a facet-level understanding of the FFM traits that were associated with each personality type. Additionally, we found that both OC and UC personality types were associated with increased eating pathology, depression, anxiety and stress, indicating that different underlying personality styles may contribute to comparable behavioural expressions of clinical disorders. Future research should focus on integrating assessment of personality and clinical conceptualising with treatment selection and outcomes in order to improve engagement in treatment for individuals presenting with clinical disorders.

### Supplementary Information

**Additional file 1:** Supplementary Documents.

## Data Availability

The datasets generated and analysed during the current study are not publicly available as ethics approval was granted under the circumstances of complete confidentiality of participant data.
