# Supplementary material for: Identifying overcontrol and undercontrol personality types among young people using the five factor model, and the relationship with disordered eating behaviour, anxiety and depression
Source: J Eat Disord. 2024 Jan 24;12:16. doi: 10.1186/s40337-024-00967-4 (PMC10809654; doi:10.1186/s40337-024-00967-4)
Supplement: Supplementary file 1 — Additional file 1: Supplementary Documents. [file 40337_2024_967_MOESM1_ESM.docx]

**Identifying Overcontrol and Undercontrol coping styles among young people using the Five Factor Model, and the relationship with disordered eating behaviour**

**Supplementary Documentation**

Contents

[Preliminary analyses 2](#_Toc153445198)

[JASP Machine Learning parameters 4](#_Toc153445199)

[Two cluster solutions 6](#_Toc153445200)

[Three cluster solutions 9](#_Toc153445201)

# Preliminary analyses

Table 1

*Means and Standard Deviations for age, EPSI, IPIP-NEO and EDE-QS by Gender, and Comparative t-tests Between Males and Females*

| Variable | |  | | Total  N = 573 | | Males  N = 167 | | Females  N = 394 | | Gender Diverse  N = 10 | |
| --- | --- | --- | --- | --- | --- | --- | --- | --- | --- | --- | --- |
|  | |  | | M | SD | M | SD | M | SD | M | SD |
| Age | |  | | 22.14 | 3.84 | 21.76 | 3.618 | 22.307 | 3.94 | 21.80 | 3.048 |
| EPSI | |  | |  |  |  |  |  |  |  |  |
|  | Body Dissatisfaction | | | 14.73 | 7.56 | 10.58 | 7.00 | 16.54 | 7.09 | 12.10 | 7.22 |
|  | Restricting | | | 8.53 | 6.22 | 7.30 | 5.86 | 9.07 | 6.29 | 8.60 | 6.52 |
|  | Binge eating | | | 12.61 | 7.72 | 11.44 | 7.02 | 13.08 | 7.90 | 12.70 | 10.37 |
|  | Purging | | | 2.74 | 4.83 | 1.49 | 3.83 | 3.28 | 5.06 | 2.30 | 7.27 |
|  | Chew and spit | | | .31 | 0.83 | 0.23 | 0.72 | 0.36 | 0.88 | 0.10 | 0.32 |
|  | Excessive Exercise | | | 7.31 | 5.42 | 8.32 | 5.12 | 6.94 | 5.51 | 6.10 | 5.04 |
|  | Muscle Building | | | 3.65 | 4.05 | 5.52 | 4.86 | 2.88 | 3.41 | 3.20 | 2.86 |
| IPIP-NEO-120 | |  | |  |  |  |  |  |  |  |  |
|  | *Neuroticism Total* | | | 77.75 | 17.27 | 69.63 | 18.28 | 81.25 | 15.75 | 14.00 | 3.65 |
|  | N1: Anxiety | | | 14.72 | 4.06 | 12.50 | 4.51 | 15.70 | 3.46 | 9.70 | 4.19 |
|  | N2: Anger | | | 11.43 | 4.42 | 10.25 | 4.47 | 11.98 | 4.31 | 12.10 | 4.33 |
|  | N3: Depression | | | 12.44 | 4.54 | 11.28 | 4.47 | 12.95 | 4.50 | 15.30 | 3.71 |
|  | N4: Self-Consciousness | | | 14.01 | 3.40 | 12.98 | 3.47 | 14.42 | 3.28 | 12.30 | 4.81 |
|  | N5: Immoderation | | | 12.01 | 3.84 | 11.54 | 3.88 | 12.20 | 3.81 | 13.40 | 4.09 |
|  | N6: Vulnerability | | | 13.14 | 3.87 | 11.08 | 4.13 | 14.01 | 3.42 | 76.80 | 13.42 |
|  | *Extraversion Total* | | | 71.55 | 14.48 | 71.32 | 15.86 | 71.57 | 13.89 | 11.10 | 4.86 |
|  | E1: Friendliness | | | 12.31 | 3.73 | 12.85 | 3.86 | 12.08 | 3.60 | 9.60 | 5.23 |
|  | E2: Gregariousness | | | 10.47 | 4.04 | 10.57 | 4.15 | 10.42 | 3.95 | 11.80 | 2.44 |
|  | E3: Assertiveness | | | 12.38 | 3.65 | 12.51 | 3.67 | 12.34 | 3.67 | 10.90 | 2.13 |
|  | E4: Activity | | | 12.06 | 3.35 | 11.14 | 3.23 | 12.48 | 3.35 | 13.80 | 3.88 |
|  | E5: Excitement Seeking | | | 11.42 | 3.17 | 11.22 | 3.05 | 11.42 | 3.17 | 11.40 | 3.10 |
|  | E6: Cheerfulness | | | 12.89 | 3.79 | 13.03 | 3.84 | 12.84 | 3.78 | 68.60 | 12.54 |
|  | *Openness Total* | | | 81.52 | 11.71 | 79.20 | 11.95 | 82.25 | 11.44 | 15.60 | 3.44 |
|  | O1: Imagination | | | 13.98 | 3.80 | 13.77 | 3.70 | 14.01 | 3.82 | 14.50 | 2.84 |
|  | O2: Artistic Interests | | | 13.96 | 3.59 | 13.24 | 3.81 | 14.24 | 3.48 | 13.10 | 5.41 |
|  | O3: Emotionality | | | 14.65 | 3.39 | 13.04 | 3.23 | 15.35 | 3.13 | 11.80 | 2.44 |
|  | O4: Adventurousness | | | 10.66 | 2.90 | 10.98 | 3.00 | 10.49 | 2.85 | 16.20 | 3.82 |
|  | O5: Intellect | | | 14.74 | 3.40 | 15.29 | 3.39 | 14.46 | 3.37 | 16.90 | 1.52 |
|  | O6: Liberalism | | | 13.53 | 3.14 | 12.88 | 3.47 | 13.70 | 2.93 | 88.10 | 11.84 |
|  | *Agreeableness Total* | | | 93.25 | 12.76 | 89.25 | 13.53 | 94.85 | 12.06 | 13.60 | 2.27 |
|  | A1: Trust | | | 13.12 | 3.77 | 13.38 | 3.85 | 12.96 | 3.74 | 15.90 | 4.31 |
|  | A2: Morality | | | 17.19 | 2.80 | 16.69 | 2.98 | 17.42 | 2.65 | 16.70 | 2.91 |
|  | A3: Altruism | | | 16.35 | 2.83 | 15.50 | 3.18 | 16.70 | 2.59 | 15.90 | 4.28 |
|  | A4: Cooperation | | | 16.06 | 3.31 | 15.43 | 3.38 | 16.32 | 3.23 | 17.20 | 3.05 |
|  | A5: Modesty | | | 14.94 | 3.83 | 13.87 | 3.86 | 15.32 | 3.75 | 16.50 | 2.95 |
|  | A6: Sympathy | | | 15.63 | 3.07 | 14.38 | 3.37 | 16.13 | 2.78 | 95.80 | 12.42 |
|  | *Conscientiousness Total* | | | 84.88 | 15.01 | 84.32 | 14.82 | 85.24 | 15.19 | 13.40 | 2.12 |
|  | C1: Self-Efficacy | | | 14.62 | 3.01 | 14.57 | 3.40 | 14.67 | 2.85 | 11.10 | 4.48 |
|  | C2: Orderliness | | | 13.16 | 4.24 | 13.42 | 4.05 | 13.11 | 4.31 | 16.40 | 1.65 |
|  | C3: Dutifulness | | | 16.16 | 2.76 | 16.00 | 2.63 | 16.21 | 2.84 | 13.90 | 2.81 |
|  | C4: Achievement Striving | | | 14.69 | 3.44 | 14.04 | 3.74 | 14.98 | 3.30 | 11.70 | 2.83 |
|  | C5: Self-Discipline | | | 12.16 | 3.36 | 12.14 | 3.44 | 12.18 | 3.35 | 13.50 | 3.57 |
|  | C6: Cautiousness | | | 14.08 | 4.04 | 14.16 | 3.88 | 14.08 | 4.14 | 80.00 | 12.30 |
| DASS | |  | |  |  |  |  |  |  |  |  |
|  | *DASS Total* | | | 51.41 | 31.30 | 46.86 | 32.80 | 53.56 | 30.48 | 17.20 | 13.34 |
|  | Depression | | | 17.47 | 12.79 | 16.74 | 12.91 | 17.88 | 12.74 | 18.20 | 12.91 |
|  | Anxiety | | | 15.26 | 11.52 | 13.95 | 11.95 | 15.79 | 11.28 | 19.60 | 8.63 |
|  | Stress | | | 18.76 | 10.83 | 16.17 | 11.10 | 19.89 | 10.59 | 55.00 | 30.71 |
| EDE-QS TOTAL | | |  | 12.06 | 8.94 | 9.28 | 7.36 | 13.34 | 9.26 | 9.00 | 10.22 |

*Note.* M = Mean, SD = Standard deviation, *df* = degrees of freedom. **p*<0.05, ***p*<0.001

# JASP Machine Learning parameters

Figure 1

Hierarchical clustering parameters


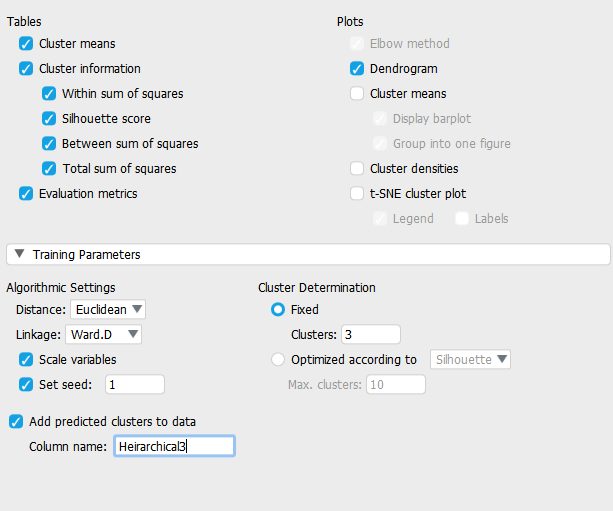


Figure 2

K-means clustering parameters


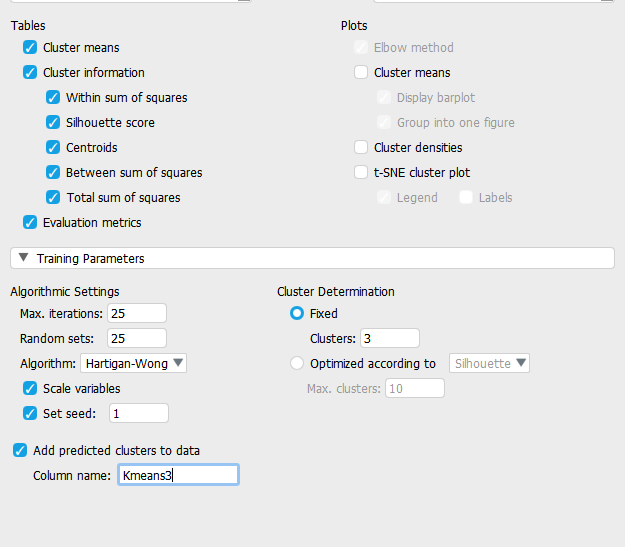


Figure 3

Random Forrest clustering parameters


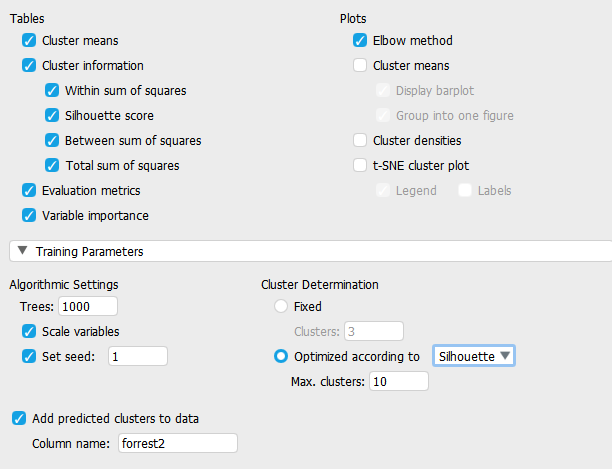


# Two cluster solutions

Table 2

Statistical Summary of Two Cluster Solutions

|  | Cluster 1  N | Cluster 2  N | R² | AIC | BIC | Silhouette |
| --- | --- | --- | --- | --- | --- | --- |
| Hierarchical | 348 | 224 | 0.108 | 13860.05 | 14094.91 | 0.100 |
| K-Means | 314 | 258 | 0.144 | 13789.88 | 14033.43 | 0.130 |
| Random Forest | 458 | 114 | 0.096 | 14569.43 | 14812.98 | 0.090 |

*Note.* Each model is optimized with respect to the *silhouette*value

Figure 4

Dendogram Depicting Hierarchical Cluster Solutions


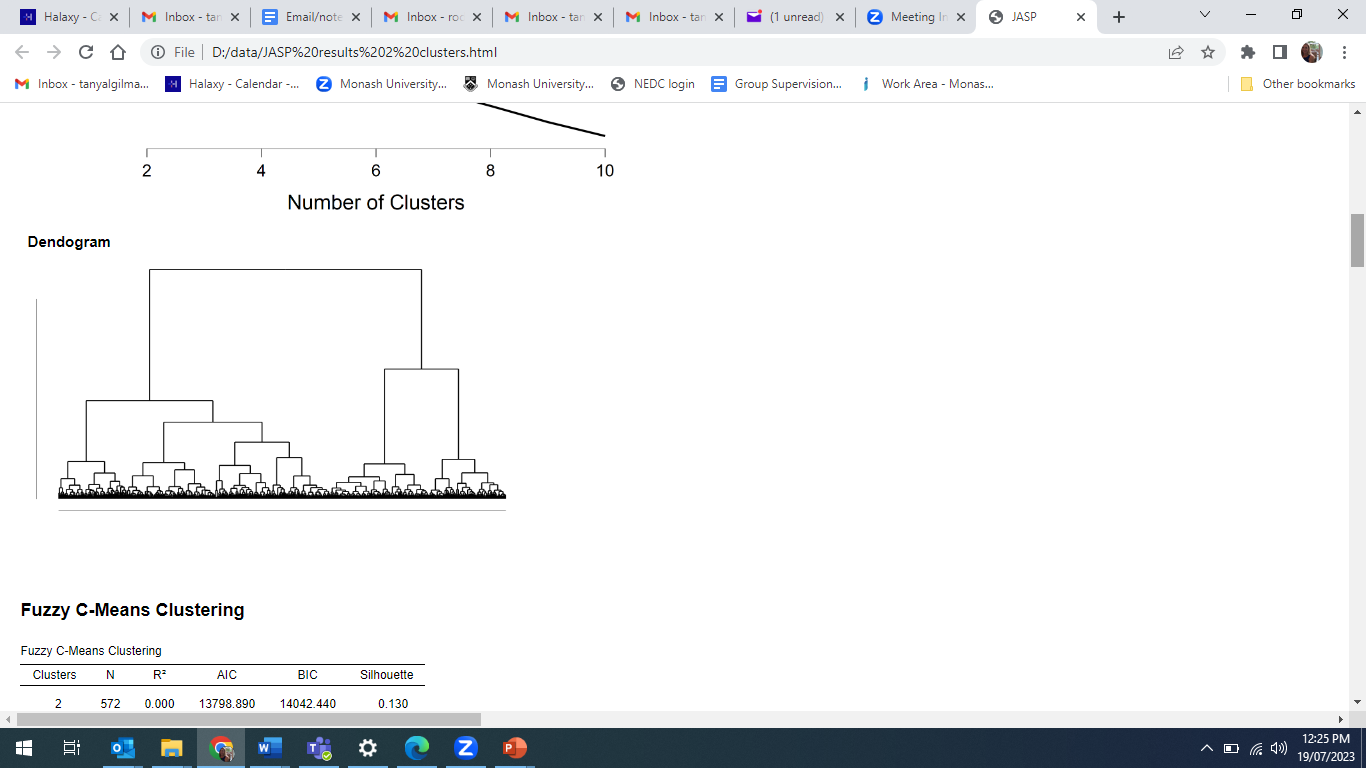


*Note*. Line indicates a three-cluster solution

Table 3

Means, Standard Deviations and independent samples t-tests results for the IPIP-NEO-120 facets, EPSI, EDE-QS and DASS-21 by cluster for all three cluster solutions

|  | Heirarchical | | | | | K-means | | | | | Random forest | | | | |
| --- | --- | --- | --- | --- | --- | --- | --- | --- | --- | --- | --- | --- | --- | --- | --- |
|  | Cluster 1  N = 348 | | Cluster 2  N = 224 | |  | Cluster 1  N = 314 | | Cluster 2  N = 258 | |  | Cluster 1  N = 458 | | Cluster 2  N = 114 | |  |
|  | M | SD | M | SD | t(df) | M | SD | M | SD | t(df) | M | SD | M | SD | t(df) |
| **IPIP-NEO-120 Neuroticism** | | | | | | | | | | | | | | | |
| N1: Anxiety | **16.52** | 2.99 | 11.94 | 3.94 | 14.85 (384.42)** | **16.30** | 3.27 | 12.80 | 4.11 | -11.09 (485.84)** | **15.81** | 3.47 | 10.35 | 3.25 | 15.22(570)** |
| N2: Anger | **12.29** | 4.46 | 10.10 | 4.01 | 6.10 (511.09)** | **13.06** | 4.23 | 9.46 | 3.80 | -10.70 (565.48)** | **12.06** | 4.45 | 8.91 | 3.21 | 8.61 (233.66)** |
| N3: Depression | **14.53** | 3.74 | 9.18 | 3.68 | 16.82 (570)** | **14.73** | 3.57 | 9.65 | 4.02 | -15.80 (519.03)** | **13.70** | 4.02 | 7.38 | 2.57 | 20.67 (267.58)** |
| N4: Self-Consciousness | **15.44** | 2.83 | 11.78 | 3.01 | 14.73 (570)** | **15.15** | 2.93 | 12.62 | 3.43 | -9.39 (508.08)** | **14.84** | 3.07 | 10.65 | 2.51 | 15.22 (205.20)** |
| N5: Immoderation | **12.83** | 3.96 | 10.73 | 3.27 | 6.92 (536.26)** | **13.45** | 3.57 | 10.26 | 3.42 | -10.83 (570)** | **12.47** | 3.89 | 10.17 | 3.03 | 6.82 (215.33)** |
| N6: Vulnerability | **15.07** | 2.98 | 10.13 | 3.12 | 19.02 (570)** | **15.02** | 3.10 | 10.85 | 3.48 | -14.98 (520.34)** | **14.24** | 3.33 | 8.70 | 2.51 | 19.63 (222.85)** |
| **IPIP-NEO-120 Extraversion** | | | | | | | | | | | | | | | |
| E1: Friendliness | 10.73 | 3.35 | **14.73** | 2.87 | -15.25 (526.03)** | 10.60 | 3.21 | **14.36** | 3.23 | 13.90 (570)** | 11.34 | 3.41 | **16.12** | 2.12 | -18.76 (276.85)** |
| E2: Gregariousness | 9.17 | 3.76 | **12.46** | 3.61 | -10.38 (570)** | 9.37 | 3.73 | **11.78** | 4.00 | 7.43 (570)** | 9.74 | 3.88 | **13.32** | 3.32 | -9.07 (570)** |
| E3: Assertiveness | 11.31 | 3.62 | **14.06** | 3.03 | -9.82 (532.34)** | 11.05 | 3.54 | **14.01** | 3.09 | 10.66 (567.79)** | 11.83 | 3.62 | **14.65** | 2.82 | -9.01 (216.12)** |
| E4: Activity | 11.43 | 3.44 | **13.04** | 2.95 | -5.96 (524.95)** | 10.99 | 3.13 | **13.36** | 3.14 | 8.98 (570)** | 11.66 | 3.37 | **13.68** | 2.73 | -6.72 (207.91)** |
| E5: Excitement Seeking | 11.13 | 3.35 | **11.85** | 2.80 | -2.76 (532.67)* | 11.57 | 3.33 | 11.22 | 2.94 | -1.36 (567.20) | 11.36 | 3.27 | 11.61 | 2.69 | -0.86 (205.03) |
| **IPIP-NEO-120 Openness** | | | | | | | | | | | | | | | |
| O1: Imagination | **14.51** | 3.79 | 13.14 | 3.66 | 4.28 (570)** | **14.38** | 3.77 | 13.47 | 3.77 | -2.87 (570)** | **14.37** | 3.78 | 12.37 | 3.43 | 5.15 (570)** |
| O2: Artistic Interests | 13.99 | 3.62 | 13.89 | 3.54 | 0.33 (570) | 13.37 | 3.55 | **14.67** | 3.52 | 4.37 (570)** | 13.88 | 3.60 | 14.26 | 3.55 | -1.03 (570) |
| O3: Emotionality | **15.09** | 3.21 | 13.94 | 3.54 | 4.04 (570)** | 14.49 | 3.37 | 14.83 | 3.41 | 1.18 (570) | 14.73 | 3.39 | 14.27 | 3.37 | 1.30 (570) |
| O5: Intellect | 14.26 | 3.50 | **15.49** | 3.10 | -4.41 (516.15)** | 13.67 | 3.41 | **16.04** | 2.91 | 9.01 (569.19)** | 14.45 | 3.46 | **15.90** | 2.89 | -4.62 (201.25)** |
| O6: Liberalism | 13.61 | 3.08 | 13.39 | 3.23 | 0.82 (570) | 13.44 | 3.02 | 13.63 | 3.28 | 0.70 (570) | 13.61 | 3.12 | 13.19 | 3.22 | 1.27 (570) |
| **IPIP-NEO-120 Agreeableness** | | | | | | | | | | | | | | | |
| A1: Trust | 12.13 | 3.76 | **14.62** | 3.23 | -8.46 (524.96)** | 11.84 | 3.60 | **14.64** | 3.35 | 9.57 (570)** | 12.58 | 3.71 | **15.22** | 3.17 | -7.68 (197.29)** |
| A2: Morality | 17.37 | 2.63 | 16.91 | 3.03 | 1.85 (427.27) | 16.39 | 2.99 | **18.16** | 2.20 | 8.10 (563.24)** | 17.11 | 2.86 | 17.49 | 2.54 | -1.29 (570) |
| A3: Altruism | 16.37 | 2.67 | 16.31 | 3.06 | 0.25 (428.63) | 15.49 | 2.93 | **17.40** | 2.30 | 8.71 (568.88)** | 16.16 | 2.88 | **17.12** | 2.45 | -3.63 (198.71)** |
| A4: Cooperation | 16.02 | 3.37 | 16.11 | 3.23 | -0.31 (570) | 14.91 | 3.41 | **17.45** | 2.58 | 10.12(566.06) ** | 15.82 | 3.44 | **17.01** | 2.54 | -4.15 (227.41)** |
| A5: Modesty | **16.05** | 3.67 | 13.20 | 3.43 | 9.29 (570)** | **15.46** | 3.86 | 14.29 | 3.71 | -3.69 (570)** | **15.40** | 3.85 | 13.06 | 3.16 | 6.75 (204.90)** |
| A6: Sympathy | **15.89** | 2.89 | 15.21 | 3.28 | 2.52 (432.11)* | 15.09 | 3.08 | 16.28 | 2.93 | 4.71 (570)** | 15.67 | 3.01 | 15.44 | 3.30 | 0.73 (570) |
| **IPIP-NEO-120 Conscientiousness** | | | | | | | | | | | | | | | |
| C1: Self-Efficacy | 13.59 | 2.94 | **16.22** | 2.34 | -11.84 (544.55)** | 13.02 | 2.73 | **16.57** | 2.02 | 17.88 (564.32)** | 14.00 | 2.95 | **17.12** | 1.60 | -15.31 (327.06)** |
| C2: Orderliness | 12.58 | 4.46 | **14.08** | 3.71 | -4.37 (533.70)** | 11.92 | 4.18 | **14.69** | 3.80 | 8.23 (570)** | 12.86 | 4.33 | **14.42** | 3.63 | -3.95 (200.86)** |
| C3: Dutifulness | 15.98 | 2.84 | 16.43 | 2.62 | -1.93 (570) | 15.03 | 2.88 | **17.52** | 1.85 | 12.51 (540.30)** | 15.84 | 2.86 | **17.40** | 1.86 | -7.10 (262.44)** |
| C4: Achievement Striving | 13.88 | 3.49 | **15.94** | 2.98 | -7.54 (527.01)** | 13.03 | 3.12 | **16.71** | 2.66 | 15.00 (570)** | 14.16 | 3.46 | **16.82** | 2.41 | -9.57 (243.38)** |
| C5: Self-Discipline | 10.93 | 3.22 | **14.08** | 2.60 | -12.85 (541.63)** | 10.40 | 2.74 | **14.31** | 2.76 | 16.94 (570)** | 11.42 | 3.18 | **15.15** | 2.28 | -14.34 (235.17)** |
| C6: Cautiousness | 13.63 | 4.33 | **14.79** | 3.45 | -3.55 (544.52)** | 12.61 | 4.13 | **15.89** | 3.11 | 10.82 (565.88)** | 13.73 | 4.21 | **15.52** | 2.89 | -5.34 (246.95)** |
| **EPSI** | | | | | | | | | | | | | | | |
| Body Dissatisfaction | **16.93** | 7.36 | 11.28 | 6.51 | 9.61 (516.57)** | **17.17** | 7.02 | 11.72 | 7.10 | -9.19 (570)** | **15.91** | 7.38 | 9.93 | 6.26 | 8.78 (198.73)** |
| Binge Eating | **13.75** | 8.14 | 10.80 | 6.63 | 4.74 (539.21)** | **14.77** | 7.70 | 9.95 | 6.87 | -7.91 (566.14)** | **13.35** | 8.00 | 9.54 | 5.52 | 5.97 (245.20)** |
| Cognitive Restraint | 5.57 | 3.56 | 5.14 | 3.23 | 1.50 (508.46) | 5.54 | 3.46 | 5.24 | 3.41 | -1.02 (570) | **5.63** | 3.57 | 4.50 | 2.69 | 3.74 (222.76)** |
| Purging | **3.43** | 5.40 | 1.65 | 3.55 | 4.76 (569.74)** | **3.84** | 5.60 | 1.40 | 3.23 | -6.52 (514.98)** | **3.21** | 5.18 | 0.83 | 2.28 | 7.37 (419.29)** |
| Restriction | **9.40** | 6.31 | 7.22 | 5.83 | 4.14 (569)** | **9.64** | 6.17 | 7.22 | 6.01 | -4.72 (569)** | **9.26** | 6.21 | 5.67 | 5.37 | 6.19 (195.59)** |
| Excessive Exercise ^Ϯ^ | 7.13 | 5.48 | 7.61 | 5.31 | -1.03 (569) | 7.23 | 5.36 | 7.44 | 5.49 | .46 (569) | 7.37 | 5.49 | 7.13 | 5.14 | 0.42 (569) |
| Negative Attitudes Towards Obesity | 6.35 | 5.59 | 6.68 | 5.40 | -0.70 (570) | **7.16** | 5.55 | 5.66 | 5.36 | -3.26 (570)* | 6.49 | 5.53 | 6.44 | 5.46 | 0.09 (570) |
| Muscle Building | 3.43 | 3.96 | 4.00 | 4.18 | -1.67 (570) | 3.83 | 4.22 | 3.44 | 3.84 | -1.15 (570) | 3.66 | 4.03 | 3.61 | 4.17 | 0.11 (570) |
| Chewing and Spitting | 0.34 | 0.83 | 0.28 | 0.82 | 0.78 (570) | **0.42** | 0.91 | 0.19 | 0.70 | -3.49 (567.47)* | **0.37** | 0.88 | 0.11 | 0.50 | 4.17 (307.09)** |
| **EDE-QS and DASS-21** | | | | | | | | | | | | | | | |
| DASS-21 Depression^Ŧ^ | **21.70** | 12.32 | 10.97 | 10.61 | 11.05 (521.79)** | **23.38** | 11.83 | 10.35 | 9.98 | -14.25 (566.67)** | **20.18** | 12.26 | 6.65 | 8.46 | 13.78 (242.59)** |
| DASS-21 Anxiety | **18.37** | 11.09 | 10.46 | 10.52 | 8.50 (570)** | **19.67** | 10.89 | 9.91 | 9.92 | -11.20 (563.91)** | **17.21** | 11.42 | 7.47 | 8.22 | 10.39 (234.18)** |
| DASS-21 Stress | **21.89** | 10.18 | 13.96 | 10.01 | 9.16 (570)** | **22.76** | 10.04 | 13.95 | 9.73 | -10.59 (570)** | **20.75** | 10.53 | 10.88 | 8.06 | 10.96 (219.44)** |
| EDE-QS ^Ŧ^ | **61.91** | 28.96 | 35.27 | 27.72 | 10.89 (567)** | **65.77** | 28.01 | 34.12 | 25.85 | -14.00 (559.69)** | **58.10** | 29.71 | 24.74 | 21.91 | 13.41 (225.85)** |

**p* <0.05, ** *p* <0.001

^Ϯ^ ANOVA is F(2,568). ^Ŧ^ ANOVA is F(2,566). Note. Matching subscripts within the same row denote significant differences. Bold denotes highest value (p<0.05).

# Three cluster solutions

Table 4

Means, Standard Deviations and one way ANOVA results for the IPIP-NEO-120 facets, EPSI, EDE-QS and DASS-21 by cluster for the Hierarchical and Random forest cluster analyses three cluster solutions

|  | Hierarchical | | | | | | | | | | | Random forrest | | | | | | | |
| --- | --- | --- | --- | --- | --- | --- | --- | --- | --- | --- | --- | --- | --- | --- | --- | --- | --- | --- | --- |
|  | OC  N = 348 | | | Resillient  N = 126 | | | UC  N = 98 | | ANOVA | | UC  N = 149 | | | OC  N = 332 | | Resillient  N = 91 | | ANOVA | |
| Subscale | M | SD | M | | SD | M | | SD | F(2,569) | Partial eta^2^ | M | | SD | M | SD | M | SD | F(2,569) | Partial eta^2^ |
| **IPIP-NEO-120 Neuroticism** | | | | | | | | | | | | | | | | | | | |
| N1: Anxiety | **16.52** ^§^ **^¶^** | 2.99 | 12.04 ^§^ | | 4.18 | 11.81 **^¶^** | | 3.64 | 124.04** | 0.30 | 14.13^§^ **^¶^** | | 3.47 | **16.38**^§#^ | 3.24 | 9.64**^¶^**^#^ | 3.06 | 154.70** | 0.35 |
| N2: Anger | **12.29**^§^ **^¶^** | 4.46 | 9.41^§#^ | | 4.06 | 10.99 **^¶^**^#^ | | 3.79 | 21.75** | 0.07 | **12.60**^§^ | | 3.77 | **11.75^¶^** | 4.65 | 8.37^§^**^¶^** | 2.98 | 30.84** | 0.10 |
| N3: Depression | **14.53**^§^ **^¶^** | 3.74 | 8.60^§#^ | | 3.70 | 9.92**^¶^**^#^ | | 3.54 | 146.44** | 0.34 | 12.70^§^ **^¶^** | | 3.75 | **13.82**^§#^ | 4.21 | 6.95**^¶^**^#^ | 2.20 | 115.01** | 0.29 |
| N4: Self-Consciousness | **15.44**^§^ **^¶^** | 2.83 | 11.71^§^ | | 3.05 | 11.87**^¶^** | | 2.97 | 108.44** | 0.28 | 13.66^§^ **^¶^** | | 2.97 | **15.16**^§#^ | 3.05 | 10.38**^¶^**^#^ | 2.55 | 94.65** | 0.25 |
| N5: Immoderation | **12.83**^§^ **^¶^** | 3.96 | 10.63^§^ | | 3.18 | 10.86**^¶^** | | 3.39 | 22.09** | 0.07 | **13.09**^§^ **^¶^** | | 3.24 | 12.14^§#^ | 4.07 | 9.77**^¶^**^#^ | 2.92 | 23.27** | 0.08 |
| N6: Vulnerability | **15.07**^§^ **^¶^** | 2.98 | 10.08^§^ | | 3.29 | 10.19**^¶^** | | 2.91 | 180.56** | 0.39 | 13.03^§^ **^¶^** | | 3.16 | **14.55**^§#^ | 3.34 | 8.19**^¶^**^#^ | 2.40 | 144.35** | 0.34 |
| **IPIP-NEO-120 Extraversion** | | | | | | | | | | | | | | | | | | | |
| E1: Friendliness | 10.73^§^ **^¶^** | 3.35 | **15.35**^§#^ | | 2.67 | 13.94**^¶^**^#^ | | 2.93 | 116.21** | 0.29 | 11.77^§^ | | 2.82 | 11.38**^¶^** | 3.65 | **16.52**^§^**^¶^** | 2.06 | 92.63** | 0.25 |
| E2: Gregariousness | 9.17^§^ **^¶^** | 3.76 | **12.52**^§^ | | 3.74 | **12.38^¶^** | | 3.46 | 53.82** | 0.16 | 10.76^§^ **^¶^** | | 3.49 | 9.40^§#^ | 3.93 | **13.84^¶^**^#^ | 3.21 | 51.58** | 0.15 |
| E3: Assertiveness | 11.31^§^ **^¶^** | 3.62 | **14.52**^§^ | | 2.90 | **13.48^¶^** | | 3.10 | 47.59** | 0.14 | 11.80^§^ | | 3.08 | 12.03**^¶^** | 3.81 | **14.67**^§^**^¶^** | 3.04 | 22.97** | 0.08 |
| E4: Activity | 11.43^§^ | 3.44 | **13.77**^§^**^¶^** | | 2.82 | 12.10**^¶^** | | 2.87 | 24.45** | 0.08 | 10.97^§^ **^¶^** | | 2.68 | 12.05^§#^ | 3.54 | **13.88^¶^**^#^ | 2.79 | 23.05** | 0.08 |
| E5: Excitement Seeking | 11.13^§^ | 3.35 | 11.20**^¶^** | | 2.67 | **12.68**^§^**^¶^** | | 2.76 | 9.86** | 0.03 | **12.68**^§^ **^¶^** | | 2.81 | 10.77^§#^ | 3.27 | 11.68**^¶^**^#^ | 2.63 | 20.37** | 0.07 |
| **IPIP-NEO-120 Openness** | | | | | | | | | | | | | | | | | | | |
| O1: Imagination | **14.51**^§^ **^¶^** | 3.79 | 13.32^§^ | | 3.72 | 12.91**^¶^** | | 3.59 | 9.47** | 0.03 | **14.52^§^** | | 3.23 | **14.31^¶^** | 3.96 | 11.84^§^**^¶^** | 3.32 | 18.35** | 0.06 |
| O2: Artistic Interests | 13.99^§^ **^¶^** | 3.62 | **15.02**^§#^ | | 3.61 | 12.44**^¶^**^#^ | | 2.89 | 15.07** | 0.05 | 12.99^§^ | | 3.09 | **14.34^§^** | 3.73 | 14.11 | 3.56 | 7.55* | 0.03 |
| O3: Emotionality | **15.09^§^** | 3.21 | **15.52^¶^** | | 2.88 | 11.91^§^**^¶^** | | 3.27 | 45.29** | 0.14 | 13.43^§^ | | 3.02 | **15.32^§¶^** | 3.34 | 14.14**^¶^** | 3.54 | 18.27** | 0.06 |
| O5: Intellect | 14.26^§^ | 3.50 | **16.66**^§^**^¶^** | | 2.70 | 13.98**^¶^** | | 2.93 | 28.57** | 0.09 | 13.77^§^ **^¶^** | | 3.20 | 14.85^§#^ | 3.47 | **15.91^¶#^** | 3.03 | 12.08** | 0.04 |
| O6: Liberalism | 13.61^§^ **^¶^** | 3.08 | **14.54**^§#^ | | 3.06 | 11.92**^¶^**^#^ | | 2.83 | 20.95** | 0.07 | 13.11^§^ | | 3.09 | 13.80 | 3.08 | **13.20^§^** | 3.36 | 3.08* | 0.01 |
| **IPIP-NEO-120 Agreeableness** | | | | | | | | | | | | | | | | | | | |
| A1: Trust | 12.13^§^ | 3.76 | **15.89**^§^**^¶^** | | 2.58 | 12.99**^¶^** | | 3.25 | 55.26** | 0.16 | 12.48^§^ | | 3.12 | 12.77**^¶^** | 3.91 | **15.36**^§^**^¶^** | 3.32 | 21.28** | 0.07 |
| A2: Morality | 17.37^§^ **^¶^** | 2.63 | **18.54**^§#^ | | 1.93 | 14.82**^¶^**^#^ | | 2.91 | 61.10** | 0.18 | 14.70^§^**^¶^** | | 2.67 | **18.20**^§^ | 2.16 | **17.57^¶^** | 2.61 | 112.61** | 0.28 |
| A3: Altruism | 16.37^§^ **^¶^** | 2.67 | **18.11**^§#^ | | 1.66 | 14.00**^¶^**^#^ | | 2.90 | 73.14** | 0.21 | 14.17^§^**^¶^** | | 2.57 | **17.10^§^** | 2.48 | **17.20^¶^** | 2.57 | 75.90** | 0.21 |
| A4: Cooperation | 16.02^§^ **^¶^** | 3.37 | **17.87**^§#^ | | 2.22 | 13.84**^¶^**^#^ | | 2.90 | 47.734** | 0.14 | 13.74^§^**^¶^** | | 3.06 | **16.75^§^** | 3.11 | **17.29^¶^** | 2.52 | 60.33** | 0.18 |
| A5: Modesty | **16.05**^§^ **^¶^** | 3.67 | 13.56^§^ | | 3.29 | 12.74**^¶^** | | 3.57 | 44.68** | 0.14 | 13.68^§^ | | 3.54 | **15.95**^§^**^¶^** | 3.76 | 13.27**^¶^** | 3.38 | 30.93** | 0.10 |
| A6: Sympathy | 15.89^§^ **^¶^** | 2.89 | **17.33**^§#^ | | 1.94 | 12.49**^¶^**^#^ | | 2.56 | 96.22** | 0.25 | 13.97^§^ **^¶^** | | 2.79 | **16.43**^§#^ | 2.76 | 15.40**^¶^**^#^ | 3.43 | 37.99** | 0.12 |
| **IPIP-NEO-120 Conscientiousness** | | | | | | | | | | | | | | | | | | | |
| C1: Self-Efficacy | 13.59^§^ **^¶^** | 2.94 | **16.94**^§#^ | | 1.96 | 15.30**^¶^**^#^ | | 2.48 | 76.33** | 0.21 | 13.40^§^ **^¶^** | | 2.53 | 14.43^§#^ | 3.06 | **17.32^¶^**^#^ | 1.59 | 59.62** | 0.17 |
| C2: Orderliness | 12.58^§^ | 4.46 | **14.51**^§^ | | 3.86 | 13.54 | | 3.46 | 10.33** | 0.04 | 11.46^§^ **^¶^** | | 3.68 | 13.47^§#^ | 4.42 | **14.88^¶^**^#^ | 3.44 | 21.73** | 0.07 |
| C3: Dutifulness | 15.98^§^ **^¶^** | 2.84 | **17.82**^§#^ | | 1.70 | 14.65**^¶^**^#^ | | 2.51 | 43.79** | 0.13 | 13.81^§^ **^¶^** | | 2.75 | **16.84^§^** | 2.30 | **17.51^¶^** | 1.96 | 100.82** | 0.26 |
| C4: Achievement Striving | 13.88^§^ | 3.49 | **17.17**^§^**^¶^** | | 2.27 | 14.37**^¶^** | | 3.04 | 49.80** | 0.15 | 12.55^§^ **^¶^** | | 2.47 | 15.01^§#^ | 3.54 | **17.02^¶^**^#^ | 2.38 | 61.85** | 0.18 |
| C5: Self-Discipline | 10.93^§^ **^¶^** | 3.22 | **14.68**^§#^ | | 2.46 | 13.31**^¶^**^#^ | | 2.59 | 82.71** | 0.23 | 10.88^§^ **^¶^** | | 2.28 | 11.86^§#^ | 3.47 | **15.40^¶^**^#^ | 2.35 | 66.71** | 0.19 |
| C6: Cautiousness | 13.63^§^ | 4.33 | **16.21**^§^**^¶^** | | 3.06 | 12.98**^¶^** | | 3.07 | 25.12** | 0.08 | 11.41^§^ **^¶^** | | 3.49 | **14.87^§^** | 4.01 | **15.62^¶^** | 2.88 | 53.73** | 0.16 |
| **EPSI** | | | | | | | | | | | | | | | | | | | |
| Body Dissatisfaction | **16.93**^§^ **^¶^** | 7.36 | 10.83^§^ | | 6.10 | 11.87**^¶^** | | 6.98 | 44.42** | 0.14 | **15.67**^§^ | | 6.87 | **15.81^¶^** | 7.62 | 9.16^§^**^¶^** | 5.84 | 32.44** | 0.10 |
| Binge Eating | **13.75**^§^ | 8.14 | 9.89^§^ | | 6.52 | 11.97 | | 6.63 | 12.45** | 0.04 | **14.56^§ ¶^** | | 6.90 | 12.69^§#^ | 8.29 | 9.03**^¶^**^#^ | 5.23 | 15.30** | 0.05 |
| Cognitive Restraint | 5.57 | 3.56 | 4.89 | | 3.16 | 5.47 | | 3.30 | 1.87 | 0.01 | 5.25 | | 3.43 | **5.70^§^** | 3.57 | 4.60^§^ | 2.78 | 3.85* | 0.01 |
| Purging | **3.43**^§^ | 5.40 | 1.07^§^ | | 2.75 | 2.40 | | 4.27 | 11.76** | 0.04 | **3.61^§^** | | 5.25 | **2.97^¶^** | 5.09 | 0.44^§^**^¶^** | 0.99 | 13.68** | 0.05 |
| Restriction ^Ϯ^ | **9.40**^§^ | 6.31 | 6.24^§^ | | 5.69 | 8.49 | | 5.78 | 12.44** | 0.04 | **9.51^§^** | | 5.64 | **9.17^¶^** | 6.45 | 4.70^§^**^¶^** | 4.64 | 22.41** | 0.07 |
| Excessive Exercise ^Ϯ^ | **7.13**^§^ | 5.48 | **6.51^¶^** | | 5.20 | 9.03^§^**^¶^** | | 5.15 | 6.64* | 0.02 | 7.70 | | 5.30 | 7.10 | 5.47 | 7.49 | 5.42 | 0.70 | 0.00 |
| Negative Attitudes Towards Obesity | 6.35^§^ | 5.59 | 5.55**^¶^** | | 5.42 | **8.14**^§^**^¶^** | | 5.04 | 6.47* | 0.02 | **7.70^§^** | | 5.32 | 5.90^§^ | 5.46 | 6.58 | 5.75 | 5.59* | 0.02 |
| Muscle Building | 3.43^§^ | 3.96 | 3.06**^¶^** | | 3.50 | **5.22**^§^**^¶^** | | 4.65 | 9.55** | 0.03 | 3.97 | | 4.25 | 3.44 | 3.84 | 3.90 | 4.49 | 1.09 | 0.00 |
| Chewing and Spitting | 0.34^§^ | 0.83 | 0.10^§¶^ | | 0.46 | **0.52^¶^** | | 1.09 | 7.75** | 0.03 | **0.51^§¶^** | | 0.98 | 0.29^§^ | 0.80 | 0.10**^¶^** | 0.52 | 7.61* | 0.03 |
| **EDE-QS and DASS-21** | | | | | | | | | | | | | | | | | | | |
| EDE-QS ^Ŧ^ | **13.92**^§^ **^¶^** | 9.43 | 7.81^§#^ | | 6.63 | 10.88**^¶^**^#^ | | 7.65 | 24.35** | 0.08 | **13.41^§^** | | 8.68 | **12.87^¶^** | 9.32 | 6.87^§^**^¶^** | 5.60 | 19.39** | 0.06 |
| DASS-21 Depression^Ŧ^ | **21.70**^§^ **^¶^** | 12.32 | 8.32^§#^ | | 8.58 | 14.41 | | 11.97 | 66.35** | 0.19 | **18.49^§^** | | 11.49 | **20.17^¶^** | 12.65 | 6.15^§^**^¶^** | 8.58 | 51.11** | 0.15 |
| DASS-21 Anxiety | **18.37**^§^ **^¶^** | 11.09 | 8.30^§#^ | | 9.33 | 13.22 | | 11.34 | 42.53** | 0.13 | **16.32^§^** | | 10.43 | **17.18^¶^** | 11.72 | 6.57^§^**^¶^** | 8.21 | 34.76 ** | 0.11 |
| DASS-21 Stress | **21.89**^§^ **^¶^** | 10.18 | 12.33^§#^ | | 9.44 | 16.04**^¶^**^#^ | | 10.39 | 46.19** | 0.14 | **19.19^§^** | | 10.17 | **21.12^¶^** | 10.50 | 9.58^§^**^¶^** | 7.83 | 47.36** | 0.14 |

**p* <0.05, ** *p* <0.001

^Ϯ^ ANOVA is F(2,568). ^Ŧ^ ANOVA is F(2,566). Note. Matching subscripts within the same row denote significant differences. Bold denotes highest value (p<0.05).
